# Integrated database for identifying candidate genes for *Aspergillus flavus* resistance in maize

**DOI:** 10.1186/1471-2105-11-S6-S25

**Published:** 2010-10-07

**Authors:** Rowena Y Kelley, Cathy Gresham, Jonathan Harper, Susan M Bridges, Marilyn L Warburton, Leigh K Hawkins, Olga Pechanova, Bela Peethambaran, Tibor Pechan, Dawn S Luthe, J E Mylroie, Arunkanth Ankala, Seval Ozkan, W B Henry, W P Williams

**Affiliations:** 1 Department of Biochemistry and Molecular Biology, Mississippi State University, MS, USA; 2 Department of Computer Science and Engineering, Mississippi State University, MS, USA; 3 Institute of Digital Biology, Mississippi State University, MS, USA; 4 Corn Host Plant Resistance Research Unit, USDA/ARS, Mississippi State, MS, USA; 5 Department of Biology, Villanova University, Villanova, PA, USA; 6 Life Sciences and Biotechnology Institute, Mississippi Agricultural and Forestry Experiment Station, Mississippi State University, MS, USA; 7 Department of Crop and Soil Sciences, The Pennsylvania State University, PA, USA; 8 Department of Human Genetics, Emory University of Medicine, GA, USA; 9 Department of Plant and Soil Sciences, Mississippi State University, MS, USA

## Abstract

**Background:**

*Aspergillus flavus* Link:Fr, an opportunistic fungus that produces aflatoxin, is pathogenic to maize and other oilseed crops. Aflatoxin is a potent carcinogen, and its presence markedly reduces the value of grain. Understanding and enhancing host resistance to *A. flavus* infection and/or subsequent aflatoxin accumulation is generally considered an efficient means of reducing grain losses to aflatoxin.  Different proteomic, genomic and genetic studies of maize (*Zea mays* L.) have generated large data sets with the goal of identifying genes responsible for conferring resistance to *A. flavus*, or aflatoxin.

**Results:**

In order to maximize the usage of different data sets in new studies, including association mapping, we have constructed a relational database with web interface integrating the results of gene expression, proteomic (both gel-based and shotgun), Quantitative Trait Loci (QTL) genetic mapping studies, and sequence data from the literature to facilitate selection of candidate genes for continued investigation. The Corn Fungal Resistance Associated Sequences Database (CFRAS-DB) (http://agbase.msstate.edu/) was created with the main goal of identifying genes important to aflatoxin resistance.  CFRAS-DB is implemented using MySQL as the relational database management system running on a Linux server, using an Apache web server, and Perl CGI scripts as the web interface.  The database and the associated web-based interface allow researchers to examine many lines of evidence (e.g. microarray, proteomics, QTL studies, SNP data) to assess the potential role of a gene or group of genes in the response of different maize lines to *A. flavus* infection and subsequent production of aflatoxin by the fungus.

**Conclusions:**

CFRAS-DB provides the first opportunity to integrate data pertaining to the problem of* A. flavus* and aflatoxin resistance in maize in one resource and to support queries across different datasets.  The web-based interface gives researchers different query options for mining the database across different types of experiments.  The database is publically available at http://agbase.msstate.edu.

## Background

Mycotoxins are considered to be among the most significant food contaminants because of their negative impact on public health, food security, and the national economy of many countries.  They affect a wide range of agricultural products, including cereals, nuts, and oilseeds, that are used for human food and animal feeds.  Mycotoxin contamination of susceptible commodities occurs as a result of environmental conditions in the field as well as improper harvesting, storage, and processing operations [1-3].  Mycotoxins may be carcinogenic, mutagenic, teratogenic, and immunosuppressive.  Estimated annual losses in the USA and Canada arising from the impact of mycotoxins on the feed and livestock industries are of the order of $5 billion. In developing countries, food staples are susceptible to contamination and it is likely that a significant number of human deaths are attributable to the consumption of mycotoxins [1].

Aflatoxins are an important group of mycotoxins that are produced as secondary metabolites under conducive climatic conditions by the fungi *Aspergillus flavus* Link:Fr. and *A. parasiticus* Speare [3].  The most common aflatoxins produced by these fungi are B1, B2, G1, and G2; the most potent of these is B1 [4,5]. Because aflatoxin induces animal diseases, particularly liver cancer in humans, aflatoxins are the most widely studied mycotoxins.  More than 50 countries have established or proposed regulations for controlling aflatoxin ingestion from contaminated foods and feeds [6-9].  The U.S. Food and Drug Administration [10] set a tolerance level of 20 ng g^-1^ for aflatoxin B1 in maize (*Zea mays* L.).  Grain that exceeds that level cannot be shipped via interstate commerce or used for human consumption.  As of 2003, 61 countries have imposed regulations on aflatoxin B1 levels in foodstuffs [11].  Aflatoxin contamination of corn reached epidemic proportions in the U.S. in 1977 and 1998.  In 1998, aflatoxin contamination resulted in $85 to $100 million in losses to maize producers in Texas, Louisiana, and Mississippi [12], and the combined crop loss due to aflatoxin epidemics in the southern USA during 1988, 1989, 1995, 1996, and 1998 surpassed $1 billion [13].  Because *A. flavus* infection and aflatoxin accumulation can lead to substantial economic losses and risk to human and animal health, efforts to create resistant corn varieties are ongoing. The seriousness of the problem of aflatoxin has generated large quantities of data from many breeding and genetics, fungal biology, physiological and biochemical studies, but the problem has yet to be solved.  

Genetics and breeding studies have focused on identifying lines with genetic resistance, either to fungal infection or specifically to aflatoxin accumulation [14-16], and mapping the resistance genes and moving them into susceptible corn inbred lines.  Biochemical studies have worked to identify the aflatoxin toxicity pathways and determine its effects on the physiology of the animals that eat infected grain and ways to mitigate these effects [4,5].  Genomics and proteomics studies of both the fungus and the corn plant, seek to identify pathways, proteins,  and genes involved in toxin metabolism and catabolism, to manipulate the production and reduction of aflatoxin and keep levels low in fungal-infected grains. Finally, many studies of resistance mechanisms to fungal infection and spread in plants are available, and much of this research may shed some light on the search for genes conferring resistance to *A. flavus*.   

The Corn Fungal Resistance Associated Sequences Database (CFRAS-DB) was created to integrate different data types from studies of aflatoxin resistance in maize by different researchers to allow us to more effectively investigate mechanisms, pathways, and genes that confer resistance to some maize lines, and allow the development of high yielding maize lines with resistance to *A. flavus* and/or aflatoxin.  CFRAS-DB uses a MySQL relational database management system with dynamic query and data integration to help researchers identify gene sequences with a possible role in aflatoxin accumulation resistance, using multiple lines of evidence.   This may help narrow the number of potential candidate genes that will be studied to determine or confirm function in aflatoxin accumulation resistance in maize.  Genes with the highest probability of being associated with aflatoxin accumulation resistance should be given priority for further study, including the creation of transgenic plants to test construct effect, association analysis of candidate genes with important phenotypes, or other biochemical or genetic tests of gene function.  

One of the major challenges of integrating data from different types of experiments conducted by different researchers over several different years is mapping the identifiers used in the different studies to a common set of identifiers.  MaizeSequence.org is a central resource for the recently sequenced maize genome and we use their gene model identifiers as reference identifiers.  Sequences from all experiments stored in CFRAS-DB are mapped to MaizeSequence.org sequences and the MaizeSequence identifiers serve as the primary key linking the different experiments. Data entries may represent DNA sequences, protein sequences, genetic loci (locations) on a chromosome, expression data of specific proteins or RNA sequences, or physiological or phenotypic traits linked to genetic markers at known loci.  This database supports multiple dynamic queries to find and display data for different genes and genotypes (individual maize plants). The outputs based on these queries can also be downloaded and used as input for pathway analysis programs for more evidence of a common function for genes these queries identify.

## Results and discussion

**Figure 1:**
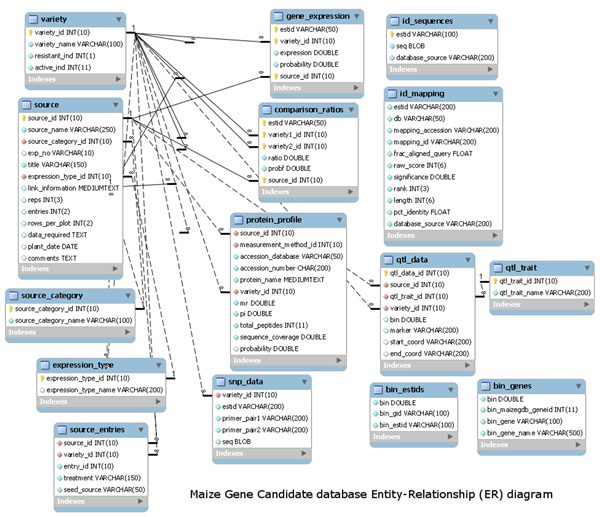
**Entity-relationship diagram for CFRAS.** This ER diagram includes the results of gene expression, proteomic, Quantitative Trait Loci (QTL) and sequence data.  These results are linked to the MaizeSequence core tables by the stable_id found in the gene_stable_id table in the MaizeSequence database.

CFRAS-DB integrates information from the newly sequenced maize genome [17] with results and annotation efforts from the aflatoxin host plant resistance community to help researchers identify genes that confer resistance in maize to *A. flavus*. Table 1 provides a list of the community datasets stored in CFRAS-DB and Table 2 provides a list of external resources integrated into CFRAS-DB. The resistance data has been collected using a wide variety of techniques, different identifiers have been used to specify genes/proteins in the experimental results from different studies, and because the newly released draft maize genome is still quite immature and will be updated on a regular basis. In addition, the resistance studies have used a variety of different maize genotypes (most are different from the B73 genotype used for the maize sequencing project) and a variety of *A.flavus* genotypes. These issues motivated a gene centric design where all entities are mapped to MaizeSequence identifiers.  This approach enables data mining across multiple experiments and easy integration with AgBase.   Figure 1 shows the entity-relationship diagram for the core CFRAS-DB tables.  

**Table 1:** Types of data currently available from the maize/*Aspergillus* research community and the type of identifier reported.

Data Type	Identifier Type	Number of Records	Data Source
Gene microarrays	ESTs with a variety of identifiers	62,178	MSU 2002 Kelley 2DAI MSU 2005 Kelley 4 DAI
Shotgun proteomics	UniProtKB	512	MSU 2004 Pechanova Rachis
Gel based proteomics	UniProtKB	411	MSU 2004 Pechanova Rachis
QTL studies	Bin numbers	67	Paul et al 2003Widstrom et al 2003Busboom 2004Unpublished QTLBrooks 2005Warburton 2009Warburton 2010
SNP studies	EST identifiers	30	MSU Mylroie SNP

**Table 2:** External data sources integrated into CFRAS-DB

External Source	Number of Records	Data Type
ZMGI	21,900	EST sequences with ids from a variety of sources
TIGR	5,071	Assembled EST sequences (TCs) with TC ids
NCBI	1,101	EST sequences and detailed gene information
MaizeGDB	25,943	Bin location for EST identifiers and genes
UniProtKB	19,006	UniProtKB protein identifiers and identifier mapping
MaizeSequence	16,955	Genome sequence, gene models, gene transcript mapping

**Figure 2:**
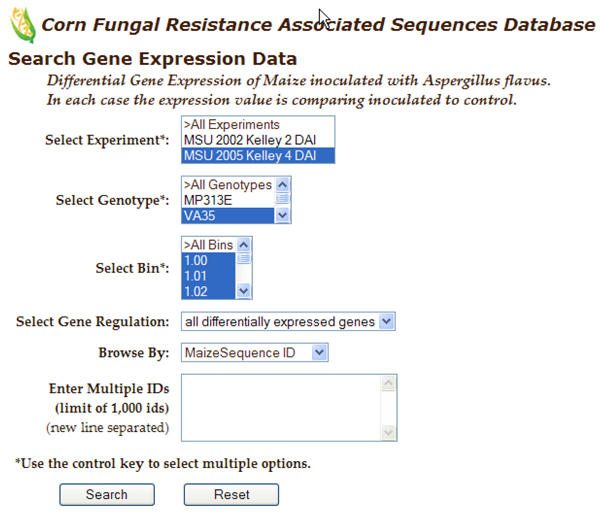
**CFRAS-DB Gene Expression query search page.** Snapshot of search interface when a specific experiment, genotype and bin(s) are selected.

**Figure 3:**
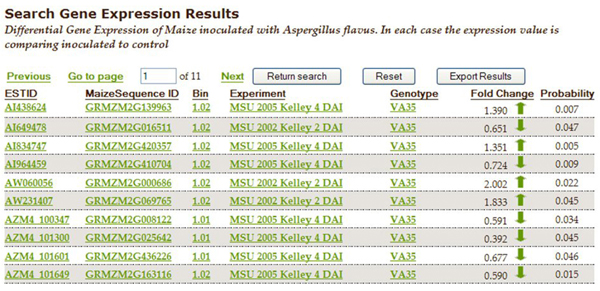
**Results generated from the query shown in Figure **2. From this results list, the user can select any ESTID, MaizeSequence ID, bin, experiment and genotype for further details.  The user can also sort the results based on any column in the results page.

**Figure 4:**
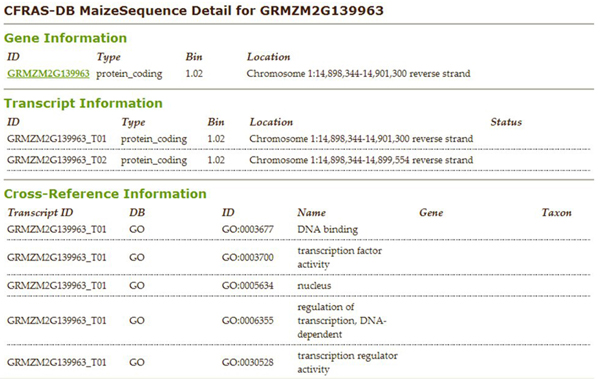
**Details generated when a gene MaizeSequence ID is selected.** Information from the MaizeSequence core tables is displayed.

The database now supports queries that enable users to identify genes implicated in resistance by multiple lines of evidence and that are the most promising for future investigations.  The web-interface provides the user with the capability to generate many additional queries.  For example, Figure 2 shows the search interface for gene expression results.   The user can select one or more experiments, genotypes, bins, direction of gene regulation, and/or specific identifiers.  Figure 3 shows the results for a gene expression query where the experiment was MSU 2005 Kelley 4 DAI, the genotype is VA35, bins are 1.00-1.02, and gene regulation is “all differentially expressed genes.”  The results page allows the user to sort the data on any column where the header is underlined (all columns in this example).  Our goal is to allow the user to access the data easily, to any desired level of detail, and from many different points of view.  If the user clicks on a sequence identifier in a gene expression list, information about the gene from MaizeSequence is displayed (Figure 4).  If the user selects an EST id, a list of all the experimental and mapping data for the corresponding MaizeSequence gene model will be displayed. From the expression results (Figure 3) one can also select a bin link.  The resulting bin data display includes QTLs that fall within the bin and a list of all sequences in the bin for which experimental data is available. From the gene expression results page, one can also explore detailed metadata about each experiment and a description of the genotypes used in the experiments. Table 3 provides a list of all queries currently available. Query capabilities will be extended and refined to meet user needs as the database expands.  CFRAS-DB results tables can be downloaded as Excel worksheets for further analysis.

**Table 3:** CFRAS-DB currently supported queries

Query Name
Gene Expression
Pairwise Genotypic Differential Expression
SNPs in Candidate Genes
QTL Study Data
Mapped QTLs by Genotype
Protein Profile Data
Combined search experiment data and gene data by genotype
Bin Contents
Combined location search for Gene Expressed data with QTL data
Gene Summary Counts

Examples of candidate genes that have been identified from multiple lines of evidence in CFRAS-DB include AW261420: Formate dehydrogenase Zea mays (Va35), AW438153: DRE binding factor 1 (dbf1) (Mp313E), TC239060: Pathogenesis-related protein 1 precursor (Va35), and AZM4_24463: Disease resistance protein RPM1 (Mp313E).  Sequencing has been performed on some candidates to find single nucleotide polymorphisms (SNP) and insertion / deletions (indels) in the target genes for further validation and possible use in developing markers for resistance to aflatoxin accumulation in maize.   These SNPs are available in CFRAS-DB. 

## Materials and methods

CFRAS-DB is implemented in MySQL 5.1.31-community version and resides on a Quad Core XEON processor X5355 2.66 GHz 2664 MHz running SUSE Linux. The web interface is implemented in Perl CGI.  The database contains data from the maize/*Aspergillus* research community (Table 1) and from external sources (Table 2).  Tables 1 and 2 illustrate the many different types of data that must be integrated.  Our strategy has been to develop procedures for mapping all of the identifiers/sequences to the newly released maize genome.  Procedures for remapping as the maize genome is refined have also been developed.  We map both EST and proteins sequences to the MaizeSequence sequences using Exonerate.  With a requirement of 80% or greater similarity, more than 99% of the 28,000 EST sequences and more than 99.5 % of the 715 unique protein sequences map to unique genes in the maize genome.

The strategy for mapping Expression Sequence Tag (EST) data and protein data to the maize genome is as follows:

1) Load the MaizeSequence core tables.  The version currently loaded is MySQL core_53_4a (http://ftp.maizesequence.org/current/databases/zea_mays_core_53_4a.sql.gz).

2) Map EST and protein identifiers to the maize sequence using Exonerate and requiring 80% similarity. [18].

3) Retrieve gene model annotation from the MaizeSequence core MySQL relational tables.

4) Link gene model annotations to AgBase functional annotations [19]

5) Link gene model annotations to bin using MaizeGDB bin coordinates [20].

In addition to sequence data and annotations, we also integrate extensive metadata describing the experiments including date, location, the genotypes used, number of days after inoculation when samples were collected, and the tissue used in the experiments.  Gene expression data is included for comparison of both control and inoculated samples and for comparison of different genotypes. All microarray data is accompanied with the metadata required for submission to the GEOarchive [21].

The gene centric design of CFRAS-DB is reflected in the entity-relationship diagram for the core CFRAS-DB tables (Figure 1).  The *gene_stable_id* table provides the link between the MaizeSequence data and the CFRAS-DB data.  CFRAS-DB currently consists of twenty-five tables excluding the MaizeSequence core tables.  Both EST identifiers and UniProt identifiers are mapped to MaizeSequence gene identifiers and a procedure for remapping has been established as MaizeSequence is updated. Because of the complexity of identifier mapping, we do not support automatic upload of data into the system.  We do encourage members of the corn fungal resistance community to submit data for inclusion in the database.  We accept gene expression data in the GEO format and proteomics data in the PRIDE format.  We will work with scientists to obtain all the necessary metadata.

### Data sources

#### QTL data

Analysis of quantitative trait loci (QTL) from resistant lines has successfully identified dozens of QTL, mainly from the following lines: Mp715 [22], Mp717 [23], Mp313E [24], Mp420, Tx6 [25,26], Tx601, and Oh516 [27].  Each QTL tends to express only in some environments, and each line tends to contain only some of the QTL.  Furthermore, very few of the QTL have a large effect, although the combined effect of several QTL can confer a high degree of resistance on a line.  Some QTL that are constant over different sources or those expressed in multiple environments can be found, especially some large effect QTL on chromosome 4 (bins 4.06 and 4.08).  Data from a number of QTL experiments is incorporated in CFRAS-DB.    

### Gene expression studies of maize (*Zea mays* L.) ears

The maize inbred lines Va35, Mp313E were used in the 2 days after inoculation (DAI) [28] microarray study while the inbred lines Va35, B73, Mp313E and Mp04:86 were used in the 4DAI microarray study. Va35 and B73 are inbred lines susceptible to aflatoxin and, Mp313E and Mp04:86 are inbred lines resistant to aflatoxin. 

The slides used were the maize Unigene [29] 1-1.05 arrays purchased from the National Science Foundation (NSF) Maize Gene Discovery Project (MGDP).  All recommendations of the minimum requirements for a microarray experiment (MIAME) checklist [30] were followed. 

Tools from AgBase (http://www.agbase.msstate.edu) and SAS Version 9.1.3 (SAS, Cary, North Carolina) were used for analysis of the data and gene ontology (GO) annotation for the differentially expressed transcripts [19]. The Agbase GoSlimViewer tool was also used to produce high-level summaries of the annotations using the Plant Go Slim available from the Gene Ontology website (http://www.geneontology.org/GO.slims.shtml).

### Proteome profile of the developing maize (*Zea mays* L.) rachis

Rachis from 21-day-old field-grown maize genotype Mp313E was profiled. For 2-DE rachis proteins were extracted with modified phenol-based protocol [31] and separated via 2-DE on 24-cm NL pH gradient 3-10 IPG strips (BioRad, Hercules, CA) and large format gels (25 cm x 20.5 cm x l.5 mm) slab gels with 10-15% polyacrylamide gradient.  Gel pots were trypsin-digested, analyzed via 1-D LC ESI MS/MS and proteins were identified against UniProt database using ProteoIQ 1.3.01 (Bioinquire, Athens GA) software. For 2-D LC, rachis proteins were extracted using 3 procedures: modified phenol extraction [31], extraction from cell debris with chaotrops and CHAPSO and DDF (Differential Detergent Fractionation) [32]. Complex protein mixtures were in-solution digested with trypsin and analyzed via 2-D LC ESI MS/MS. Proteins were identified using ProteoIQ 1.3.01 (Bioinquire) software and UniProtKB database [33].

### Single Nucleotide Polymorphisms

DNA was extracted using the CTAB method from frozen, freeze-dried leaf tissue.  DNA was extracted from multiple resistant and susceptible lines. Primers were designed for each gene by using the EST sequence obtained from NCBI (http://www.ncbi.nlm.nih.gov/).  Primer 3 software v. 0.40 (http://frodo.wi.mit.edu/primer3/) was used to design the primer pairs, and primers were ordered from Sigma – Genosys.  Primers were verified using PCR amplification and PCR products were visualized on a 1.5% agarose gel stained with ethidium bromide. 

PCR products were purified using Qiagen QIAquickTM PCR Purification Kit (Qiagen Inc.; Valencia, CA), and sequencing reactions were prepared using Big Dye® chemistry from Applied Biosystems Inc. (Foster City, CA). The sequencing reactions were then analyzed using an Applied Biosystems 3730xl DNA Analyzer.  Sequencing was performed on multiple resistant and susceptible maize genotypes, and alignment of sequences was performed using DNAMAN software v. 5.2.9 (Lynnon Corporation; Pointe-Claire, Quebec, Canada).

## Future directions and conclusions

We are in the process of integrating three additional proteomics datasets and an RNASeq dataset into CFRAS-DB.  The addition of RNASeq data will significantly increase the size of the database.  We plan to develop a prioritization algorithm that will rank genes by the number of experiments where they were found to be differentially expressed between resistant and susceptible lines, that map to QTL known to be involved in aflatoxin accumulation resistance, have been reported in the literature to be involved in aflatoxin accumulation resistance, and that contain genes or domains known to be involved in pathways that do not favor the production of aflatoxin. 

The proteins and gene sequences identified via the queries can be investigated with pathway analyses to find larger patterns indicating major mechanisms of resistance to fungal infection or aflatoxin accumulation in maize.  The reports generated by the queries can be formatted for automatic update into network analysis programs.  In addition to maize genetics, genomics, proteomics, and biochemical studies, data from different *A. flavus* strains can be added, and perhaps resistance data from maize infected with other fungal strains as well.  Information on environment data (from field phenotyping studies) will allow more complexity to be added to the analyses, which is important considering the complexity of the trait under study.

The CFRAS database and the associated web-based interface allow researchers to examine many lines of evidence to assess the potential role of a gene or group of genes in the response of different maize lines to *A. flavus* infection and subsequent production of the toxin, aflatoxin, by the fungus. CFRAS contains data from QTL experiments, gene expression studies, proteomics studies (both gel-based and shotgun), as well as sequence data from the literature. The database also contains results by other research groups who have identified genes potentially involved in resistance or susceptibility to *A. flavus*, and we expect more will follow. The web-based interface provides researchers with the capability to mine the database across many different experiments.

### Availability and requirements

The database is publicly available from the AgBase main page (http://www.agbase.msstate.edu).  Researchers wishing to submit data to CFRAS-DB should contact cfrasdb@cse.msstate.edu.  Access to the restricted database can be made available to submitters prior to publication of their results to facilitate analysis.  

## Authors' contributions

RYK facilitated the discussion on the database, led the writing effort, helped develop the requirements of the database, and conducted the gene expression studies.  JWH designed and created the initial database.  MLW helped design the structure of the database, co-wrote and edited the article, and did the QTL studies and meta-analysis.  CG and SMB designed the database structure, developed the id mapping approach   implemented the database and the web-based interface, and co-wrote the article.  LKH helped design the structure of the database, co-wrote and edited the article.  OP and TP performed proteomic analysis of maize rachis and contributed to writing the article. BP performed proteomic analysis of maize silk.  DSL led the proteomic studies and provided guidance. JEM aided in gene expression studies and conducted the SNP studies. AKA aided in gene expression studies, and contributed to data extraction. SO reviewed published literature and manually curated the references to select candidate genes. WBH helped specify the requirements for the database and edited the manuscript. WPW initiated, planned, and oversaw the QTL and gene expression studies and assembled the group of investors working on the database project.

## Competing interests

The authors declare that they have no competing interests.
